# The Interaction Between Genetic Variant ZNF804A rs1344706 and Alcohol Withdrawal on Impulsivity: Evidence for the Diathesis-Stress Model

**DOI:** 10.3389/fpsyt.2021.761237

**Published:** 2022-01-03

**Authors:** Xie Zhang, Huankun Sun, Fan Wang, Michelle Niculescu, Guanghui Shen, Siyao Zhou, Fan Yang, Yu-Hsin Chen, Li Chen, Wei Wang, Yanlong Liu

**Affiliations:** ^1^Department of Pharmacy, Ningbo Medical Treatment Center, Li Huili Hospital, Ningbo, China; ^2^School of Mental Health, Wenzhou Medical University, Wenzhou, China; ^3^Psychiatry Research Center, Beijing Hui-Long-Guan Hospital, Peking University, Beijing, China; ^4^Xinjiang Key Laboratory of Neurological Disorder Research, The Second Affiliated Hospital of Xinjiang Medical University, Urumqi, China; ^5^Department of Social Sciences, Chatham University, Pittsburgh, PA, United States; ^6^Department of Psychology, College of Liberal Arts, Wenzhou-Kean University, Wenzhou, China; ^7^The Affiliated Kangning Hospital, Wenzhou Medical University, Wenzhou, China

**Keywords:** ZNF804A, alcohol dependence, impulsivity, single nucleotide polymorphism, GxE

## Abstract

**Objective:** Alcohol use disorder (AUD) is the most common substance use disorder, which may relate to increased impulsivity. A more detailed understanding of the potential moderating factor on association between AUD and impulsivity is likely to have far-reaching effects. This study aims to examine whether the interaction between a genetic variant ZNF804A rs1344706 and alcohol use is related to impulsivity in Chinese Han adult males diagnosed with AUD.

**Methods:** A total of 455 Chinese Han adult males diagnosed with AUD were included in this study. Impulsivity was assessed using Barratt Impulsiveness Scale. Alcohol dependence was measured by Michigan Alcoholism Screening Test. Genomic DNA was extracted from peripheral blood of participants and genotyped.

**Results:** Hierarchical multiple regression yielded a significant interaction between ZNF804A rs1344706 and alcohol use (β = 0.20, *p* = 0.0237). Then, A region of significance (RoS) test was performed to interpret the interaction effect. Re-parameterized regression models revealed that the interaction between ZNF804A rs1344706 and alcohol problem severity fit to the weak diathesis-stress model (*R*^2^ = 0.15, *p* < 0.0010), indicating that the T allele carriers are more susceptible to alcohol problem severity, jointly contributing to impulsivity.

**Conclusions:** This study, which analyzed a specific gene-environment interaction, demonstrated that carriers of the T allele of ZNF804A rs1344706 may be more susceptible to alcohol problem severity, correlated with higher levels of impulsivity during withdrawal.

## Introduction

Alcohol use disorders (AUDs) including the more severe forms of alcohol dependence and withdrawal are the most common substance use disorders, raising severe public concerns worldwide (1, 2). The estimated global prevalence of AUDs was 5.1% in 2016 (3). In mainland China, the prevalence of AUDs for males reached 10.1% (4), which leads to a high burden of physical conditions as well as psychological disorders. AUDs can be characterized as an adaptive state formed by repeated alcohol use, which can lead to withdrawal upon cessation. During withdrawal, the reward and stress systems result in continued use in relation to long-term neurocognitive changes. The neurocognitive consequences of AUDs include impairments in memory, attention, and executive function (5–7), which may result in risky decision-making and lack of planning or focusing, culminated in an increase in impulsivity. Paradoxically, impulsivity has also been recognized as a risk factor for excessive alcohol use (8). Therefore, a more detailed understanding of the relationship between AUDs and impulsivity is likely to have far-reaching effects in terms of prevention and treatment.

However, the presence and extent of impulsivity under the context of alcohol withdrawal, varies greatly among individuals, which could not be fully explained by external stressors alone. A meta-analysis of twin, family, and adoption studies demonstrated that the genetic influences could explain approximately half of the variance in impulsivity (9). Thus, genetic vulnerability that may influence the environmental contributors on impulsivity has attracted more attention. One study showed that participants with the DRD2 C957T polymorphism demonstrated more reward-related impulsivity following a psychosocial stressor (10). Moreover, another group found that five polymorphisms related to the dopaminergic and serotonergic signaling systems contributed to impulsivity, with some variants interacting with early adversity (11). These findings highlight the importance of examining genetic influences that interact with environmental pressures (gene and environment interaction, G×E interaction). However, similar studies have focused on the genes encoding neuronal signaling-related proteins, while upstream regulatory genes are rarely involved.

ZNF804A, a gene that reached the genome-wide significance for schizophrenia (12) and encodes the protein that directly contributes to transcriptional control of schizophrenia associated gene PRSS16 and COMT (13), raised our interest. It is reported that the ZNF804A rs1344706 single nucleotide polymorphism (SNP) is associated with impaired decision-making in those that abuse heroin (14). In patients with chronic schizophrenia, ZNF804A rs1344706 C/G genotype were associated with impulsivity (15). However, ZNF804A is the gene that encodes zinc finger protein 804A, which is involved in multiple cellular processes such as DNA-binding and protein interactions. Because it is related to multiple complex biological functions, no single specific pathway can be targeted. The role of the SNP ZNF804A rs1344706 in the formation and progression of psychosis is still unclear.

To date, few studies have examined the exact form of the interaction between the environment and ZNF804A rs1344706 gene polymorphism. Existing studies on G×E interactions can be fit into two possible models, diathesis-stress model, and differential susceptibility model, to explain the possible role of genetic factors (16). The diathesis-stress model hypothesizes that individuals with risk genes are more likely to be affected by adverse environmental factors, jointly contributing to psychological problems. While the differential susceptibility model also describes that under the influence of a certain genetic characteristic, individuals who are susceptible to the negative environmental effects will show psychological or behavioral problems, it also includes the converse genetic susceptibility to positive environmental factors, exhibiting better outcomes.

This study aims to examine the role of ZNF804A rs1344706 on impulsivity in patients actively withdrawing while suffering from moderate to severe AUD. Specifically, the form of G×E interaction based on two theoretical models (diathesis-stress or differential susceptibility) is identified using confirmatory analytic approaches. According to the current knowledge of ZNF804A rs1344706, T allele carriers tend to be susceptible to psychosis. Therefore, we hypothesized that ZNF804A rs1344706 would conform to the diathesis-stress model, anticipating carriers of ZNF804A rs1344706 risk allele would show more impulsivity than individuals with the low-risk allele during alcohol withdrawal (poor environment conditions).

## Methods

### Participants and Genotyping

In this study, 452 Chinese male participants were recruited from six hospitals including Mental Health Center in Shandong Provincial, the Sixth Hospital in Changchun, Mental Health Center in Shenyang, the Third Hospital in Inner Mongolia Autonomous Region, Mental Health Center in Hulunbuir, Mental Health Center in Tongliao, covering most part of northern China. Primary inclusion criteria for the alcohol dependent participants were as follows: (1) diagnosed with alcohol dependence by at least two trained psychiatrists according to DSM-IV; (2) aged from 18-65; (3) are Han Chinese. Participants who meet the following conditions were excluded: (1) have a history of other substance addiction (excluding nicotine); (2) diagnosed with severe cardiovascular, hepatic, or renal conditions; (3) patients or first-degree relatives with a history of severe mental illness; (4) inability to understand the informed consent form. Demographic data including age and years of education were collected. Genotyping was performed as previously described (17). Briefly, genomic DNA was extracted from 5 ml peripheral blood of each participant with salting-out method, and the ZNF804A rs1344706 SNP was genotyped using MALDI-TOF based scalable MassARRAY System (Agena Bioscience, Inc., San Diego, CA, United States) with PCR primers: 5-TCAAAGCCTTATCTCTTCAC-3, 5-CCAGATAGATATCCAAGAAG-3, and single-base extension primer: 5-ACTGAAACAAAGAATCAAAAAC-3 for genotyping. Ten percent of the DNA samples were duplicated randomly and tested, and no error was found.

### Measures

#### Alcohol Problem Severity

Alcohol problem severity was measured by the Michigan Alcoholism Screening Test (MAST) (18). The MAST is a 25-item, self-report questionnaire on which respondents rate the severity of a range of alcohol use behaviors related to dependence using a 4-point scale ranging from 1 (not at all) to 4 (extremely). The scale has high internal-consistency reliability, with alpha values of 0.90 (19). Higher scores indicate greater alcohol problem severity.

#### Impulsivity

The Barratt Impulsiveness Scale (BIS) (20) is a questionnaire designed to assess the personality/behavioral construct of impulsiveness. It is a 30-item questionnaire that covers three second-order factors of impulsiveness including attentional impulsiveness, motor impulsiveness, and non-planning impulsiveness. It is the most widely cited instrument for the assessment of impulsiveness and has a high internal-consistency reliability, with alpha values of 0.80 (21). Higher scores indicate higher impulsivity.

#### Data Analysis

First, the Hardy–Weinberg equilibrium for genotype distributions of ZNF804A rs1344706 was tested using the χ^2^ test. Pearson's correlation was conducted to examine the associations among genetic polymorphisms, age, year of education, MAST, and BIS scores. Consistent with other research, GT and TT genotypes were collapsed into the T-allele group and coded as 1 and the GG genotype was coded as 0.

Second, traditional linear regression was used to provide initial testing for G×E interaction. When significant interactions were founded, we used RoS analysis for interaction effects. This approach provides the lower and higher bound where the association between gene and alcohol dependence is significant for estimating the forms of G×E interaction. Finally, re-parameterized regression model was fitted to test the nature of G×E interactions (16), which had the form:


$$\text{Y=}\{\begin{array}{c} \text{Group}:\text{D = 0 }{\text{B}}_{\text{0}}{\text{ + B}}_{\text{1}}\left(\text{X}-\text{C}\right){\text{ + B}}_{\text{3}}{\text{X}}_{\text{2}}{\text{ + B}}_{\text{4}}{\text{X}}_{\text{3}}\text{ + E}\\ \text{Group}:\text{D = 1 }{\text{B}}_{\text{0}}{\text{ + B}}_{\text{2}}\left(\text{X}-\text{C}\right){\text{ + B}}_{\text{3}}{\text{X}}_{\text{2}}{\text{ + B}}_{\text{4}}{\text{X}}_{\text{3}}\text{ + E} \end{array}$$

Here Y is the dependent variable of BIS score, group was allelic group, X was MAST score, X_2_ and X_3_ was covariates: age and years of education. C was the intersection point at which the slopes for the two gene groups cross. What distinguishes the diathesis-stress and differential susceptibility models is the location of the crossover point C. If the point estimate and 95% confidential interval estimate falls at the maximum of alcohol addiction, the interaction is consistent with diathesis-stress model. Conversely, if the estimate of C is within the range of alcohol addiction, the forms of interaction are consistent with differential susceptibility model.

These two models can be further subdivided into “strong/weak diathesis-stress model” and “strong/weak differential susceptibility model.” Strong versions assume that only “risk/plasticity allele” carriers are susceptible to environment, while the weak versions assume that both allele carriers are susceptible to environment but “non-risk/non-plasticity allele” carriers are less susceptible to environment than “risk/plasticity allele” carriers. These models are nested within each other. An *F*-test was conducted to compare model if one model include parameter estimates than another one. For non-nested models, we compare Akaike information criterion (AIC) and Bayesian information criterion (BIC) to evaluate which model fits better.

## Results

### Descriptive Analysis (Correlation of MAST and BIS Scores)

Descriptive statistics of research variables were shown in Table 1. The genotypic distribution of ZNF804A rs1344706 in 452 patients was as follows: 108 (23.89 %) GG homozygotes, 235 (46.81%) GT heterozygotes, and 109 (24.12 %) TT homozygotes, which was consistent with Hardy–Weinberg equilibrium (χ^2^ = 0.72, *p* > 0.0500), as shown in Table 2.

**Table 1:** Descriptive statistics.

**Variables**	**Mean (SD)**
Age	44.11 (9.25)
Education years	10.78 (2.84)
Impulsivity	119.56 (45.28)
MAST score	9.15 (5.49)

*Impulsivity is measured by Barratt Impulsiveness Scale (BIS), alcohol problem severity is measured by Michigan Alcoholism Screening Test (MAST)*.

**Table 2:** Hardy-Weinberg equilibrium.

**Genotype**	**Number of people**	**Percentage**	
GG	108	23.89%	
GT	235	51.99%	
TT	109	24.12%	
χ^2^	0.72	*p-value*	0.3971

The correlations among variables are shown in Table 3. No significant correlation between variant ZNF804A rs1344706 and MAST or BIS scores were observed. BIS scores were positively correlated with MAST scores (*r* = 0.37, *p* < 0.0010), while the years of education was negatively correlated with both MAST (*r* = −0.24, *p* < 0.0010) and BIS scores (*r* = −0.20, *p* < 0.0010).

**Table 3:** Descriptive statistics and correlations among study variables.

	**rs1344706**	**Age**	**Education years**	**Alcohol problem severity**	**Impulsivity**
Rs1344706	1				
Age	0.04	1			
Education years	0.01	−0.39^***^	1		
Alcohol problem severity	0.01	0.21^***^	−0.24^***^	1	
Impulsivity	−0.03	0.03	−0.20^***^	0.37^***^	1

*Impulsivity is measured by Barratt Impulsiveness Scale (BIS), alcohol problem severity is measured by Michigan Alcoholism Screening Test (MAST).*

*** *p < 0.0010*.

Participants were grouped according to their genotype of ZNF804A rs1344706 into GG homozygotes and T allele carriers. Independent sample *t*-test showed no difference between genotypic groups in terms of age, years of education, MAST, and BIS scores (Table 4).

**Table 4:** Independent sample test.

**rs1344706 polymorphism**	**Age**	**Education years**	**Alcohol problem severity**	**Impulsivity**
GG homozygote	43.64 (9.18)	10.70 (3.03)	9.40 (5.60)	119.26 (50.16)
T allele	44.25 (9.27)	10.81 (2.78)	9.07 (5.47)	119.66 (43.72)
*T*	−0.60	−0.32	0.54	−0.08
*P*	0.5476	0.7463	0.5917	0.9364

*Impulsivity is measured by Barratt Impulsiveness Scale (BIS), alcohol problem severity is measured by Michigan Alcoholism Screening Test (MAST)*.

### The Interactions of Alcohol Dependence and ZNF804A rs1344706 on Impulsivity

**Figure 1:**
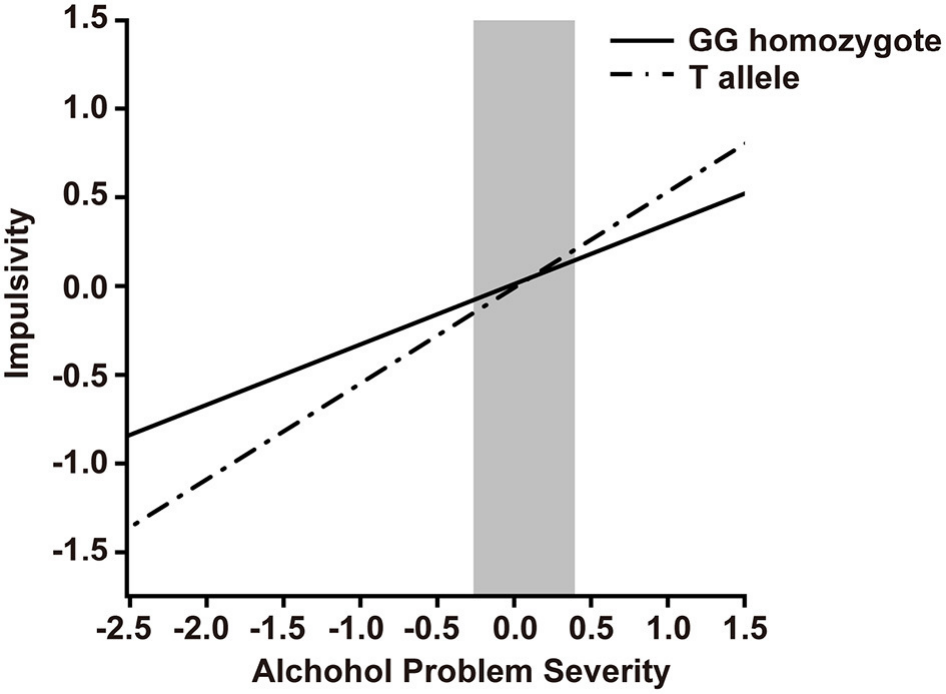
RoS test on Impulsivity from Alcohol Problem Severity in rs1344706 allelic groups. Impulsivity is measured by Barratt Impulsiveness Scale (BIS), alcohol problem severity is measured by Michigan Alcoholism Screening Test (MAST). Gray shaded area represents 95% CI of the crossover point C of the interaction on the alcohol dependence axis. 95% CI of C ranged from−0.26 to 0.39. Simple slope at GG homozygote = 0.34, *t* = 10.75, *p* < 0.0010. Simple slope at T allele = 0.54, *t* = 11.51, *p* < 0.0010.

To identify the interaction between ZNF804A rs1344706 genotype and alcohol problem severity on impulsivity, we conducted hierarchical multiple regression analysis (Table 5). Both alcohol problem severity (β = 0.34, *p* < 0.0010) and education (β = 0.20, *p* < 0.0010) showed main effects on BIS scores, while the ZNF804A rs1344706 genotype showed no main effect (β = −0.01, *p* = 0.7450) on BIS scores. In the next step, the interaction of alcohol problem severity and ZNF804A rs1344706 were included in the equation, and explained significant additional variance (β = 0.20, *p* = 0.0237). A region of significance (RoS) test was performed to interpret the interaction effect (Figure 1). The slopes for alcohol dependence on impulsivity were as follows: GG homozygote β = 0.34, *t* = 10.75, *p* < 0.0010; T allele carriers β = 0.54, *t* = 11.51, *p* < 0.0010, suggesting that the T allele carriers showed more alcohol problem severity and tended to be more impulsive.

**Table 5:** Interaction between rs1344706 and Alcohol problem severity on impulsivity.

	**Variables**	**Impulsivity**					
		***ΔR^2^***	***B*** **(*SE*)**	**β**	***T***	***p-*value**	**95%CI**
Step1	Education Years	0.04	0.07 (0.02)	0.20	4.39	<0.0010	[0.04, 0.10]
Step2	Alcohol problem severity	0.11	0.34 (0.04)	0.34	7.56	<0.0010	[0.25, 0.43]
	rs1344706		−0.03 (0.10)	−0.01	−0.33	0.7450	[-0.23, 0.17]
Step3	Alcohol problem severity × rs1344706	0.01	0.23 (0.10)	0.20	2.27	0.0237	[0.03, 0.43]

*Impulsivity is measured by Barratt Impulsiveness Scale (BIS), alcohol problem severity is measured by Michigan Alcoholism Screening Test (MAST)*.

### Internal Replication Analysis

We employed re-parameterized regression models to test the robustness of the interaction and examine the specific fit model of G×E interaction. As seen in Table 6, Model D explained a significant amount of variance in impulsivity, *R*^2^ = 0.15, *p* < 0.0010. The crossover point C = 1.57, is fixed to the maximum of alcohol problem severity, providing support for the weak diathesis-stress model. Furthermore, we conducted an F test and compared the AIC and BIC values to test if Model D is better fit than Model A, B, and C. Constraining B1 = 0 led to Model C (strong diathesis-stress model). Relaxing the fixed C led to Model B (weak differential susceptibility model). Both constraining B1 = 0 and relaxing the C point, led to Model A (strong differential susceptibility model). As is shown in Table 6, Model C explained less variance (*R*^2^ = 0.12, *p* < 0.0010) than that of Model D, leading us to reject Model C. Compared with Model D, Model B has one more parameter but its ΔR^2^ is not significantly increased (ΔR^2^ = 0.01, *p* > 0.0500), resulting in the rejection of Model B. Moreover, The AIC and BIC values of Model D are relatively smaller than that of Model A, lending support for Model D (weak diathesis-stress model).

**Table 6:** Results for re-parameterized regression model for Impulsivity.

	**Differential susceptibility**		**Diathesis-stress**	
**Parameter**	**Strong:**	**Weak:**	**Strong:**	**Weak:**
	**Model A**	**Model B**	**Model C**	**Model D**
B_0_	−0.52 (0.19)	−0.44 (0.23)	−0.33 (0.19)	0.04 (0.20)
B_1_	0.00 (–)	0.17 (0.09)	0.00 (–)	0.28 (0.06)^***^
C	0.05 (0.26)	0.12 (0.45)	1.57 (–)	1.57 (–)
95%CI of C			(–)	(–)
B_2_	0.39 (0.05) ^***^	0.39 (0.05)^***^	0.26 (0.04)^***^	0.36 (0.05)^***^
B_4_	0.05 (0.02)^**^	0.04 (0.02)^**^	0.06 (0.02)^***^	0.04 (0.02)^**^
R^2^	0.15	0.16	0.12	0.15
F (df)	26.87^***^ (3,448)	28.25^***^ (4,447)	29.29^***^ (2,449)	27.10^***^ (3,448)
F vs. C (df)	19.59^***^ (1,448)	19.69^***^ (2,447)	(–)	20.21^***^ (1,448)
F vs. D (df)	(–)	2.90 (1,447)	20.21^***^ (1,447)	(–)
AIC	1216.93	1215.40	1234.28	1216.33
BIC	1237.50	1240.08	1250.74	1236.90

** *p < 0.0100;*

*** *p < 0.0010*.

## Discussion

Based on the framework of G×E research on the etiology of AUD-related psycopsychiatrical issues, this study examined the interaction between ZNF804a rs1344706 and alcohol problem severity during withdrawal on impulsivity in Han Chinese patients diagnosed with alcohol dependence. We found that the alcohol problem severity had a main effect on impulsivity and the interaction between ZNF804a rs1344706 and alcohol problem severity fit the weak diathesis-stress model, indicating that the T allele carriers are more susceptible to alcohol problem severity, jointly contributing to impulsivity.

Consistent with previous studies, we find that impulsivity was positively correlated with alcohol problem severity. Patton et al. divided impulsivity into three dimensions: attentional impulsiveness, motor impulsiveness, and non-planning impulsiveness (20). It is reported that the impulsive decision-making, a non-planning trait of impulsivity, is a predictor of alcohol use and alcohol pathology in a sample of problem drinkers (22). A recent study found alcohol use in adolescents is associated with all three domains of impulsivity (23). In our study, years of education were negatively correlated with both alcohol problem severity and impulsivity and plays a mediating role in our multiple regression model. Years of education is often related to memory (24), attention, and executive function (25), which may be the possible shared neurocognitive mechanisms impaired in AUDs and in subjects with higher impulsivity (26). Well-educated individuals may be more likely to work through problems rather than seek to drink alcohol to avoid the problem and obtain immediate rewards. Education may provide individuals with more knowledge, better critical thinking skills, and the ability to process information. In addition, better education is correlated with higher socioeconomical status, which is positively associated with material resources and health behaviors (27).

Further, to examine the effect of G×E interaction on impulsivity, hierarchical multiple regression was performed. Alcohol problem severity shows a main effect on impulsivity, and the SNP ZNF804a rs1344706 and education year showed moderating effects. The RoS test and internal replication analysis was performed to find the model that could better explain the regulatory role of ZNF804A rs1344706. The weak version of diathesis-stress model was best fitted. T allele (risk allele) carriers were more susceptible to alcohol problem severity and tended to be more impulsive in relation to alcohol problem severity during withdrawal. In accordance with the present results, the ZNF804A rs1344706 risk allele is associated with impaired decision-making in those that suffer from substance abuse (14).

Several studies have attempted to define the effect of the rs1344706 genotype on functional connectivity and morphological structure of the brain. In subjects with schizophrenia, the ZNF804A risk allele (A/T) is associated with relatively intact gray matter volume, particularly for hippocampal volumes (28). Voineskos et al. (29) found that those homozygous for the risk allele had reduced cortical gray matter thickness in the superior temporal gyrus and the anterior and posterior cingulate cortices compared with non-risk allele carriers, consistent with the findings that they showed reduced attention control, one trait of increased impulsivity. As the ZNF804A rs1344706 is an intronic SNP, the mechanism by which the “risk allele” works remains poorly understood. Studies of its role on ZNF804A expression shed a light on this issue. During fetal brain development, the T allele of rs1344706 is associated with a relative decrease in ZNF804A expression (30). Similarly, the expression of ZNF804A^E3E4^, a transcript isoform of ZNF804A abundantly expressed in the brain, is reduced in fetal brain (31), providing evidence for the mechanism of the risk allele function. Given that the ZNF804A plays an active role in neurite formation, maintenance of dendritic spines, and activity-dependent structural plasticity *in vitro* (32), the reduced expression of ZNF804A exerted by risk allele is a likely risk factor for aberrant neuronal development and is consistent with neuroimaging study findings of ZNF804A (28, 29).

Several limitations of the current study should be addressed. The main limitation of this study is that the population did not include female patients. Further, biases such as social desirability were unavoidable because the behavioral measures were self-report scales. In addition, the baseline impulsivity of participants is not measured prior to the admission and diagnosis of alcohol dependence, which greatly limited the strength of our study. An additional uncontrolled factor is the possibility that various withdrawal symptoms may contribute to impulsivity, which could be explored in further research. Finally, we did not examine the separate effects of AUD on different facets of impulsivity. In summary, the strong external validity of real-world settings of hospitalized alcohol-dependent patients limited the internal validity of these findings.

Despite limitations, the study yields several important conclusions. First, alcohol problem severity during alcohol withdrawal in men hospitalized for alcohol dependence showed a main effect on impulsivity, and the SNP ZNF804a rs1344706 showed a moderating effect. Second, T allele carriers of ZNF804a rs1344706 were more sensitive to alcohol problem severity and tended to be more impulsive during alcohol withdrawal. Detection of SNP ZNF804a rs1344706 may provide a clinic predictor of impulsivity in alcohol dependent patients. Further studies with larger sample size or the setting of specific time points should be conducted to examine the generalizability of the results of this study.

## Data Availability Statement

The raw data supporting the conclusions of this article will be made available by the authors, without undue reservation.

## Ethics Statement

The study was approved by the Ethics Committee of Peking University Health Science Center. The patients/participants provided their written informed consent to participate in this study.

## Author Contributions

XZ, HS, and FW: data collection, literature review, and wrote the first draft of the manuscript. MN, GS, SZ, and FY: wrote sections of the manuscript. GS and Y-HC: performed statistical analysis. LC, WW, and YL: contributed to conception and design of the study. All authors read and approved the final manuscript.

## Funding

This study was supported by the Natural Science Foundation of Xinjiang Province (2018D01C239) and the National College Student Innovation and Entrepreneurship Training Program (201910343031). The fund had no role in the design of this study, in the collection, analysis and interpretation of data, in the writing of the report, and in the decision to submit the paper for publication.

## Conflict of Interest

The authors declare that the research was conducted in the absence of any commercial or financial relationships that could be construed as a potential conflict of interest.

## Publisher's Note

All claims expressed in this article are solely those of the authors and do not necessarily represent those of their affiliated organizations, or those of the publisher, the editors and the reviewers. Any product that may be evaluated in this article, or claim that may be made by its manufacturer, is not guaranteed or endorsed by the publisher.
